# 828. Do lung co-infections influence outcome in patients with hematological malignancies and culture-documented invasive pulmonary aspergillosis?

**DOI:** 10.1093/ofid/ofad500.873

**Published:** 2023-11-27

**Authors:** Takahiro Matsuo, Sebastian Wurster, Ying Jiang, Jeffrey Tarrand, Dimitrios P Kontoyiannis

**Affiliations:** The University of Texas MD Anderson Cancer Center, Houston, TX; The University of Texas MD Anderson Cancer Center, Houston, TX; The University of Texas MD Anderson Cancer Center, Houston, TX; The University of Texas MD Anderson Cancer Center, Houston, TX; The University of Texas MD Anderson Cancer Center, Houston, TX

## Abstract

**Background:**

Although pulmonary co-infections are common in patients (pts) with hematological malignancies (HM) and invasive pulmonary aspergillosis (IPA), there is a paucity of recent studies on the type and the impact of co-infections on outcome in contemporary pts with HM and culture-documented IPA.

**Methods:**

We retrospectively reviewed the records of pts with HM at MD Anderson Cancer Center (January 2016 and October 2021) who had microbiologically-documented IPA (based on sputum and bronchoscopy lavage culture) to identify risk factors, clinical features, evidence of co-infections, and outcome. Independent risk factors for 42-day mortality from IPA diagnosis were assessed using a binary multivariable logistic regression model.

**Results:**

Among 128 IPA pts (19 proven, 109 probable), 48 pts (38%) had neutropenia (< 500/µL) and 38 (30%) had prior allogeneic hematopoietic cell transplant. *Aspergillus fumigatus* was the commonest agent of IPA (41%), Fig. 1). Potential co-pathogens were present in 79 pts (62%) at the time of IPA diagnosis. Bacteria (most commonly Gram-negative rods) and less commonly other fungi were isolated in 32 (25%) and 7 pts (5%), respectively (Fig. 2). In addition, a variety of upper respiratory viruses (40 pts, 31%) and cytomegalovirus (CMV, 15 pts, 12%) were recovered. 42-day mortality after IPA diagnosis was 48%, with no significant difference between pts with and without lung co-infections (48% vs 47%, p >0.99, Table 1). Acute kidney injury (adjusted odds ratio [aOR] 2.51, 95% confidence interval [CI] 1.31-5.17, p=0.003), Sequential Organ Failure Assessment score ≥ 8 (aOR 4.78, 95% CI 2.66-9.41, p=< .0001), and recovery from neutropenia (aOR 0.41, 95% CI 0.20-0.78, p=0.004) were independent predictors of 42-day mortality.

Type of Aspergillus spp (number of isolates)

**Figure:**
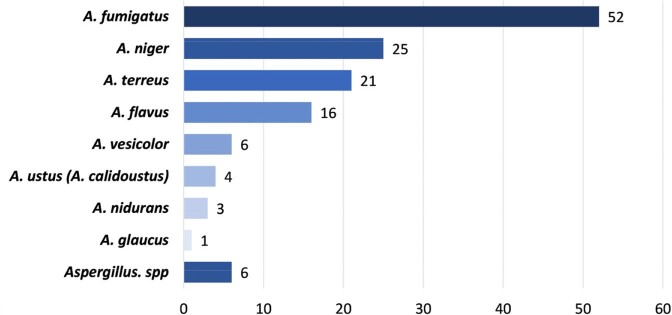

**Figure 1.**

Isolated co-pathogens (number of isolates)

**Figure:**
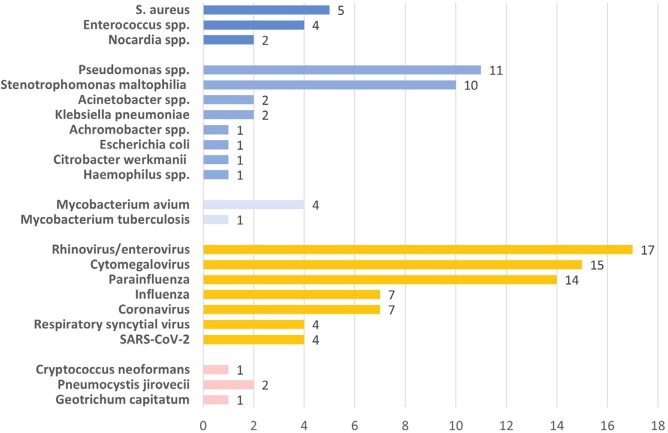

**Figure 2.**

Co-pathogens and 42-day mortality

**Figure:**
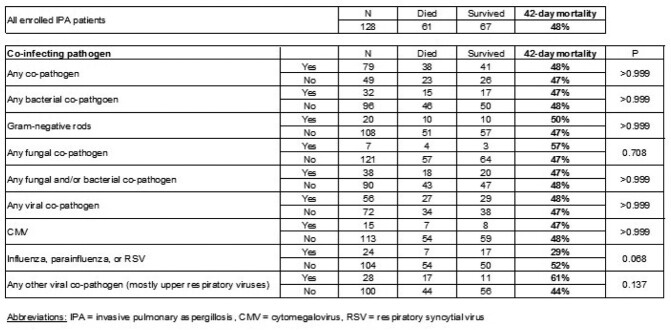

**Table 1.**

**Conclusion:**

A quarter of patients with culture-documented IPA had concurrent lung infections with bacteria (especially Gram-negative bacteria) and less commonly with fungi. In addition, co-culture of upper respiratory viruses or CMV was common. In a background of high mortality of culture-documented IPA, there was no excess mortality if a lung co-infection was present. Further studies are needed regarding the impact of inter-kingdom interactions in pts with earlier, galactomannan-based (but BAL culture negative) IPA diagnosis.

**Disclosures:**

**Dimitrios P. Kontoyiannis, MD, MS, ScD, PhD**, AbbVie: Board Member|Astellas: Grant/Research Support|Cidara: Board Member|Gilead: Grant/Research Support|Merck: Advisor/Consultant|Scynexis/MSGERC: Board Member

